# National prevalence and sociodemographic correlates of potential depression, anxiety, and suicidality among the young population in Kazakhstan: a national population-based study

**DOI:** 10.1186/s40359-026-05618-8

**Published:** 2026-10-01

**Authors:** Xinyu Li, Andrew Stickley, Yuanxuan Cai, Yilin Gai, Geir Bjørklund, Yunfei Li, Fan Jiang, Shanquan Chen

**Affiliations:** 1 Shandong Provincial ENT Hospital, Shandong University, Jinan, Shandong China; 2 Department of Preventive Intervention for Psychiatric Disorders, National Institute of Mental Health, National Center of Neurology and Psychiatry, Tokyo, Japan; 3 School of Pharmacy, Huazhong University of Science and Technology, Wuhan, China; 4 School of Public Health, Capital Medical University, Beijing, China; 5 Council for Nutritional and Environmental Medicine, Mo i Rana, Norway; 6 Department of Neurobiology, Care Sciences and Society, Karolinska Institutet, Stockholm, Sweden; 7 School of Public Health, Shandong University, Jinan, 250021 Shandong China; 8 School of Public Health, Li Ka Shing Faculty of Medicine, The University of Hong Kong, Hong Kong, China; 9 Department of Health and Rehabilitation Sciences, Faculty of Medicine and Health Sciences, Stellenbosch University, Stellenbosch, South Africa

**Keywords:** Mental health, Depression, Anxiety, Suicide, Young population, Kazakhstan

## Abstract

**Background:**

Mental health disorders are the primary driver of the global disease burden among young people, yet nationally representative data for Central Asia remains scarce. This study aimed to determine the prevalence and sociodemographic correlates of potential depression, anxiety, and suicidality among the young population in the Central Asian nation of Kazakhstan.

**Methods:**

A cross-sectional analysis was conducted using data from the 2024 Kazakhstan Multiple Indicator Cluster Survey (MICS), a nationally representative, multi-stage probability sample. Potential depression and anxiety were screened using the PHQ-2 and GAD-2 instruments (cut-off ≥ 3), while suicidality was assessed via self-reported thoughts (past two weeks) or attempts (past 12 months). Weighted prevalence estimates were calculated, and modified Poisson regression models identified independent sociodemographic risk factors. Population attributable fractions (PAFs) were estimated to quantify the impact of associated risks.

**Findings:**

We included 5,079 individuals aged 15–24 years. The weighted national prevalence was 1.70% (95%CI = 1.31–2.08) for potential depression, 1.59% (95%CI = 1.23–1.96) for potential anxiety, and 0.96% (95%CI = 0.65–1.27) for potential suicidality among the population aged 15–24 years. In adjusted analyses, female sex was the strongest predictor of both potential depression (adjusted risk ratio [aRR] = 2.72, 95%CI = 1.55–4.79) and anxiety (aRR = 2.80, 95%CI = 1.58–4.96). Non-Kazakh ethnicity was also significantly associated with an increased risk of depression (aRR = 1.88, 95%CI = 1.17–3.02) and anxiety (aRR = 1.99, 95%CI = 1.28–3.11). PAFs indicated that female sex accounted for 45.6% and 46.8% of the risk for depression and anxiety, respectively. No significant sociodemographic correlates were identified for suicidality.

**Conclusion:**

These findings provide the first nationally representative benchmarks for youth mental health in Kazakhstan. While prevalence rates are lower than global averages, the marked disparities by sex and ethnicity highlight the need for targeted, culturally sensitive mental health interventions. Strengthening early detection and resource allocation for these vulnerable subgroups is essential to meet national and Sustainable Development Goal targets.

**Supplementary Information:**

The online version contains supplementary material available at https://doi.org/10.1186/s40359-026-05618-8.

## Introduction

Adolescent mental health represents a global public health priority, as psychiatric disorders during this developmental window are primary drivers of morbidity and mortality, notably through suicidality [1]. Anxiety and depressive disorders frequently emerge during adolescence and young adulthood, often following a chronic or relapsing course if left unaddressed. Globally, approximately 300 million individuals aged 5–24 years live with a mental health disorder, contributing nearly 30 million years lived with disability (YLDs) [2]. Furthermore, suicide remains a leading cause of death among those aged 15–29 years, with 75% of these fatalities occurring in low- and middle-income countries (LMICs) [3]. In Eastern Europe and Central Asia, suicide is the foremost cause of mortality in the young population [4].

Despite the significant disease burden, a recent scoping review highlighted that mental health research is critically under-prioritized within public health systems across Central Asia [5]. To date, empirical data regarding mental health difficulties among youth aged 15–24 years in this region remain sparse [6]. Evidence suggests that youth suicide is an escalating crisis in Kazakhstan [7]; according to the United Nations Children’s Fund (UNICEF), the risk of suicidal behavior among Kazakhstani adolescents (15–19 years) is three times higher than the average across the Commonwealth of Independent States (CIS), a regional organization in Eurasia [8].

While the prevalence and sociodemographic correlates of depression and anxiety have been extensively documented in other global contexts [9], [10], these parameters remain insufficiently characterized in Kazakhstan. Existing literature has predominantly focused on adult cohorts or specific administrative regions [11–13]. For instance, a cross-sectional survey of 1,021 adults during the COVID-19 pandemic identified a 12.0% prevalence of depression and 8.4% for anxiety [14]. Another study in the Karaganda Region reported depressive symptoms in 75.7% of adults, with higher rates observed in females [13]. Untreated mental disorders are associated with profound adverse outcomes, including educational attrition, diminished economic productivity, and increased risk of incarceration or homelessness, impacting both the individual and broader society [15], [16].

Current evidence in Kazakhstan is largely derived from small-scale regional surveys, leaving a critical gap in nationally representative epidemiological data. The 2024 Kazakhstan Multiple Indicator Cluster Survey (MICS) marked a significant milestone by including a dedicated mental health module for participants aged 15–24 years. Such data are fundamental for informing evidence-based prevention strategies and achieving Sustainable Development Goal (SDG) Target 3.4, which mandates the promotion of mental health and well-being across the lifespan [17].

This study aims to investigate the prevalence and sociodemographic correlates of potential depression, anxiety, and suicidality among young people aged 15–24 years in Kazakhstan using a nationally representative sample. We specifically address two research questions: (1) What is the national prevalence of these conditions among Kazakhstani youth? and (2) Which sociodemographic factors—including age, sex, residence, education, marital status, and household wealth—independently associate with these outcomes?

## Method

### Study design and participants

This study utilized a cross-sectional, population-based design analyzing data from the 2024 Kazakhstan Multiple Indicator Cluster Survey (MICS), which was implemented by the Bureau of National Statistics of Kazakhstan with technical and financial support from UNICEF to collect critical health and development indicators. To ensure the robust tracking of Sustainable Development Goals (SDGs) and national health development, the survey employed a two-stage, stratified cluster sampling design, generating representative estimates at the national, regional, and urban-rural levels across all 17 regions and three major municipalities of Kazakhstan. In the first stage, census enumeration areas were systematically selected within urban and rural strata with probability proportional to size. In the second stage, households were systematically selected within each enumeration area. Eligible household members were interviewed using standardized questionnaires, including a household questionnaire administered at the household level and individual questionnaires administered to specific population groups (women aged 15–49 years, men aged 15–24 years, children aged < 5 years, and children aged 5–17 years). All questionnaires were administered by trained data collectors following the MICS7 protocol to ensure international comparability. Overall, 22,454 households were successfully interviewed (response rate 96.3%), with response rates of 97.4% for women and 94.4% for men aged 15–24 years.

For this study, we used data from the men’s and women’s questionnaires, restricted to individuals aged 15–24 years. Participants were excluded from the analysis if more than 50% of items were missing on either the brief depression or anxiety scales, resulting in a final analytical sample of 5,079 participants (Supplementary Fig. 1).

Verbal informed consent was obtained from all participants. For respondents aged 15–17 years, parental or guardian consent was obtained prior to the participant’s assent. A detailed description of the survey methodology can be found in the Kazakhstan MICS 2024 report, available on the UNICEF MICS website [18].

### Outcome measures

Potential depression and anxiety were evaluated using two validated brief screening instruments: the 2-item Patient Health Questionnaire (PHQ-2) and the 2-item Generalized Anxiety Disorder Scale (GAD-2), both of which are recognized as being as effective as their longer versions for rapid initial screening [19], [20]. While formal validation of these instruments in Kazakhstan is limited, evidence from systematic reviews and meta-analyses supports the reliability and validity of ultra-brief screening instruments across diverse cultural and international settings [20], [21]. The PHQ-2 assessed the frequency of depressed mood—defined by feelings of sadness and hopelessness—and anhedonia—defined as the loss of pleasure or interest in previously enjoyable activities—over the preceding two weeks. The GAD-2 evaluated feelings of being nervous, anxious, or on edge, alongside the inability to stop or control worrying. All items were rated on a four-point Likert scale ranging from 0 (not at all) to 3 (almost every day), with total scores for each scale ranging from 0 to 6; a cut-off point of ≥ 3 was used to indicate potential depression or anxiety [22]. More details about the depression and anxiety measures available in Supplementary Table 1.

Suicidality was assessed as a binary variable based on participants’ self-reported thoughts and behaviors. Participants were classified as having suicidality if they responded affirmatively to having tried to harm themselves with the intention to end their life in the past 12 months, or having thought about doing something to end their life in the past two weeks.

### Sociodemographic variables

In this study, we considered the following sociodemographic factors based on prior literature [23]. The primary independent variables included age (stratified as 15–19 and 20–24 years), sex (male or female), and area of residence (urban area or rural area). Educational attainment was categorized into high school and below or college and above, while marital status was divided into married or unmarried. Household wealth quintiles were developed by MICS for each survey using a wealth index based on principal component analysis (PCA) of household characteristics, including dwelling conditions, access to water and sanitation, and ownership of consumer goods. To ensure adequate sample sizes within each category and improve the statistical stability of the estimates, our study further classified into low, middle, and high tiers. Ethnicity was recorded as either Kazakh or non-Kazakh ethnicities. More details about these variables available in Supplementary Table 1.

## Statistical analysis

The proportion of missing data for the included variables was minimal, remaining below 1% for potential depression (0.2%), anxiety (0.1%), and suicidality (0.2%). These missing values were addressed using multiple imputation by chained equations (MICE) with five imputations performed via the ‘mice’ package in R, incorporating all independent and outcome variables to minimize bias.

Descriptive statistics were used for all outcomes, with continuous data reported as mean values and standard deviations, while categorical data were presented as frequencies and percentages. Associations between categorical variables were tested using Pearson’s chi-square test with Rao & Scott adjustment to account for the complex survey design. All analyses incorporated weighting and clustering by enumeration district using the R survey package to ensure national representativeness [24].

To identify independent risk factors, modified Poisson regression models were applied for each outcome, with results reported as adjusted risk ratios (aRRs) and 95% confidence intervals (CIs) [25]. This approach was chosen to directly estimate risk ratios, which provide more interpretable measures of association and are consistent with the estimation of population attributable fractions (PAFs). Furthermore, PAFs were calculated to quantify the potential reduction in mental health outcomes achievable through the modification of associated risk factors using the formula $\:{PAF}\mathrm{=}\frac{{p}\mathrm{(}{RR}\mathrm{-1)}}{{p}\left({RR}\mathrm{-1}\right)\mathrm{+1}}$, where p is the prevalence of the risk factor and RR is the fully adjusted risk ratio.

Weighted prevalence estimates were visually presented in plots displaying age-sex and area-sex stratified patterns and highlighting group-specific differences, with 95% CI. Prevalence by region was illustrated on maps to visualize spatial patterns of the outcome.

Sensitivity analyses were conducted on unimputed data to confirm the robustness of the primary findings.

All statistical procedures were performed using R version 4.3.2 with a significance threshold of *p* < 0.05.

## Results

### Prevalence of potential depression, anxiety, and suicidality

A total of 5,079 young individuals aged 15–24 years were included in the final analytical sample. Compared with excluded participants, included participants differed significantly in marital status (*p* < 0.001) and residence (*p* = 0.012), and no significant differences were observed in age, sex, education, wealth, and ethnicity (Supplementary Table 2). Population estimates (weighted percentages) indicated that the study population was predominantly 15–19 years (58.0%), resided in urban areas (67.6%), was comprised of unmarried individuals (85.6%), and was of Kazakh ethnicity (72.4%) (Table 1). Overall, the weighted national prevalence was 1.70% (95% CI 1.31–2.08) for potential depression, 1.59% (95% CI 1.23–1.96) for potential anxiety, and 0.96% (95% CI 0.65–1.27) for potential suicidality. In bivariate analyses, potential depression and anxiety were significantly more frequent among individuals aged 20–24 years (*p* = 0.012), females (*p* < 0.001), unmarried participants (depression: *p* = 0.004; anxiety: *p* = 0.009), and those of non-Kazakh ethnicity (depression: *p* = 0.005; anxiety: *p* = 0.002). Conversely, no significant sociodemographic differences were observed for the prevalence of potential suicidality.

**Table 1:** The characteristics of 5,079 young people aged 15–24 years in Kazakhstan

Variable	Overall unweighted	Depression			Anxiety			Suicidality		
		No depression	Depression	*p*-value	No anxiety	Anxiety	*p*-value	No suicidality	Suicidality	*p*-value
Total	5079	4985; [98.30% (97.92–98.69)	94; [1.70% (1.31–2.08)]		4985; [98.41% (98.04–98.77)]	94; [1.59% (1.23–1.96)]		5031; [99.04% (98.73–99.35)]	48; [0.96% (0.65–1.27)]	
Age				0.012			0.012			0.333
15–19 years	2891 (58.0%)	2850; [98.72% (98.29–99.14)]	41; [1.28% (0.86–1.71)]		2848; [98.78% (98.38–99.18)]	43; [1.22% (0.82–1.62)]		2869; [99.16% (98.77–99.56)]	22; [0.84% (0.44–1.23)]	
20–24 years	2188 (42.0%)	2135; [97.73% (97.02–98.43)]	53; [2.27% (1.57–2.98)]		2137; [97.89% (97.25–98.53)]	51; [2.11% (1.47–2.75)]		2162; [98.87% (98.39–99.35)]	26; [1.13% (0.65–1.61)]	
Sex				< 0.001			< 0.001			0.599
Male	1790 (51.2%)	1774; [99.10% (98.63–99.58)]	16; [0.90% (0.42–1.37)]		1775; [99.18% (98.75–99.61)]	15; [0.82% (0.39–1.25)]		1772; [98.96% (98.44–99.48)]	18; [1.04% (0.52–1.56)]	
Female	3289 (48.8%)	3211; [97.46% (96.88–98.04)]	78; [2.54% (1.96–3.12)]		3210; [97.59% (97.03–98.15)]	79; [2.41% (1.85–2.97)]		3259; [99.12% (98.78–99.47)]	30; [0.88% (0.53–1.22)]	
Residence				0.542			0.230			0.116
Urban	3115 (67.6%)	3054; [98.23% (97.73–98.73)]	61; [1.77% (1.27–2.27)]		3049; [98.26% (97.78–98.74)]	66; [1.74% (1.26–2.22)]		3092; [99.21% (98.84–99.58)]	23; [0.79% (0.42–1.16)]	
Rural	1964 (32.4%)	1931; [98.45% (97.89–99.01)]	33; [1.55% (0.99–2.11)]		1936; [98.71% (98.18–99.24)]	28; [1.29% (0.76–1.82)]		1939; [98.68% (98.10–99.27)]	25; [1.32% (0.73–1.90)]	
Education				0.845			0.498			0.142
High school and below	2848 (57.2%)	2800; [98.33% (97.79–98.88)]	48; [1.67% (1.12–2.21)]		2797; [98.51% (98.06–98.96)]	51; [1.49% (0.76–1.82)]		2815; [98.85% (98.40–99.30)]	33; [1.15% (0.70–1.60)]	
College or above	2231 (42.8%)	2185; [98.26% (97.73–98.78)]	46; [1.74% (1.22–2.27)]		2188; [98.26% (97.67–98.85)]	43; [1.74% (1.15–2.33)]		2216; [99.29% (98.90-99.67)]	15; [0.71% (0.33–1.10)]	
Marital status				0.004			0.009			0.830
		Depression, n (%)			Anxiety, n (%)			Suicidality, n (%)		
		No depression	Depression	p-value	No anxiety	Anxiety	p-value	No suicidality	Suicidality	p-value
Unmarried	4211 (85.6%)	4143; [98.54% (98.15–98.92)]	68; [1.46% (1.08–1.85)]		4141; [98.61% (98.24–98.98)]	70; [1.39% (1.02–1.76)]		4172; [99.03% (98.68–99.38)]	39; [0.97% (0.62–1.32)]	
Married	868 (14.4%)	842; [96.92% (95.56–98.28)]	26; [3.08% (1.72–4.44)]		844; [97.22% (95.96–98.48)]	24; [2.78% (1.52–4.04)]		859; [99.11% (98.47–99.74)]	9; [0.89% (0.26–1.53)]	
Wealth				0.543			0.104			0.100
Low	2920 (55.4%)	2868; [98.25% (97.71–98.80)]	52; [1.75% (1.20–2.29)]		2863; [98.32% (97.84–98.79)]	57; [1.68% (1.21–2.16)]		2885; [98.75% (98.29–99.21)]	35; [1.25% (0.79–1.71)]	
Middle	1041 (21.5%)	1023; [98.67% (98.05–99.28)]	18; [1.33% (0.72–1.95)]		1029; [99.10% (98.58–99.62)]	12; [0.90% (0.38–1.42)]		1034; [99.39% (98.87–99.92)]	7; [0.61% (0.08–1.13)]	
High	1118 (23.1%)	1094; [98.08% (97.23–98.93)]	24; [1.92% (1.07–2.77)]		1093; [97.97% (97.01–98.93)]	25; [2.03% (1.07–2.99)]		1112; [99.41% (98.92–99.90)]	6; [0.59% (0.10–1.08)]	
Ethnicity				0.005			0.002			0.716
Kazakh	3738 (72.4%)	3681; [98.65% (98.26–99.04)]	57; [1.35% (0.96–1.74)]		3684; [98.76% (98.38–99.14)]	54; [1.24% (0.86–1.62)]		3703; [99.08% (98.73–99.43)]	35; [0.92% (0.57–1.27)]	
Non-Kazakh	1341 (27.6%)	1304; [97.38% (96.41–98.35)]	37; [2.62% (1.65–3.59)]		1301; [97.48% (96.59–98.36)]	40; [2.52% (1.64–3.41)]		1328; [98.94% (98.26–99.62)]	13; [1.06% (0.38–1.74)]	

Data are n (unweighted); Weighted percentages (%) were calculated using survey weights, with 95% confidence intervals (CI); % are calculated with row totals as the denominator. P-values were calculated based on Pearson’s χ² test with Rao & Scott adjustment for categorical variables

### Sex and age-specific variations

**Fig. 1:**
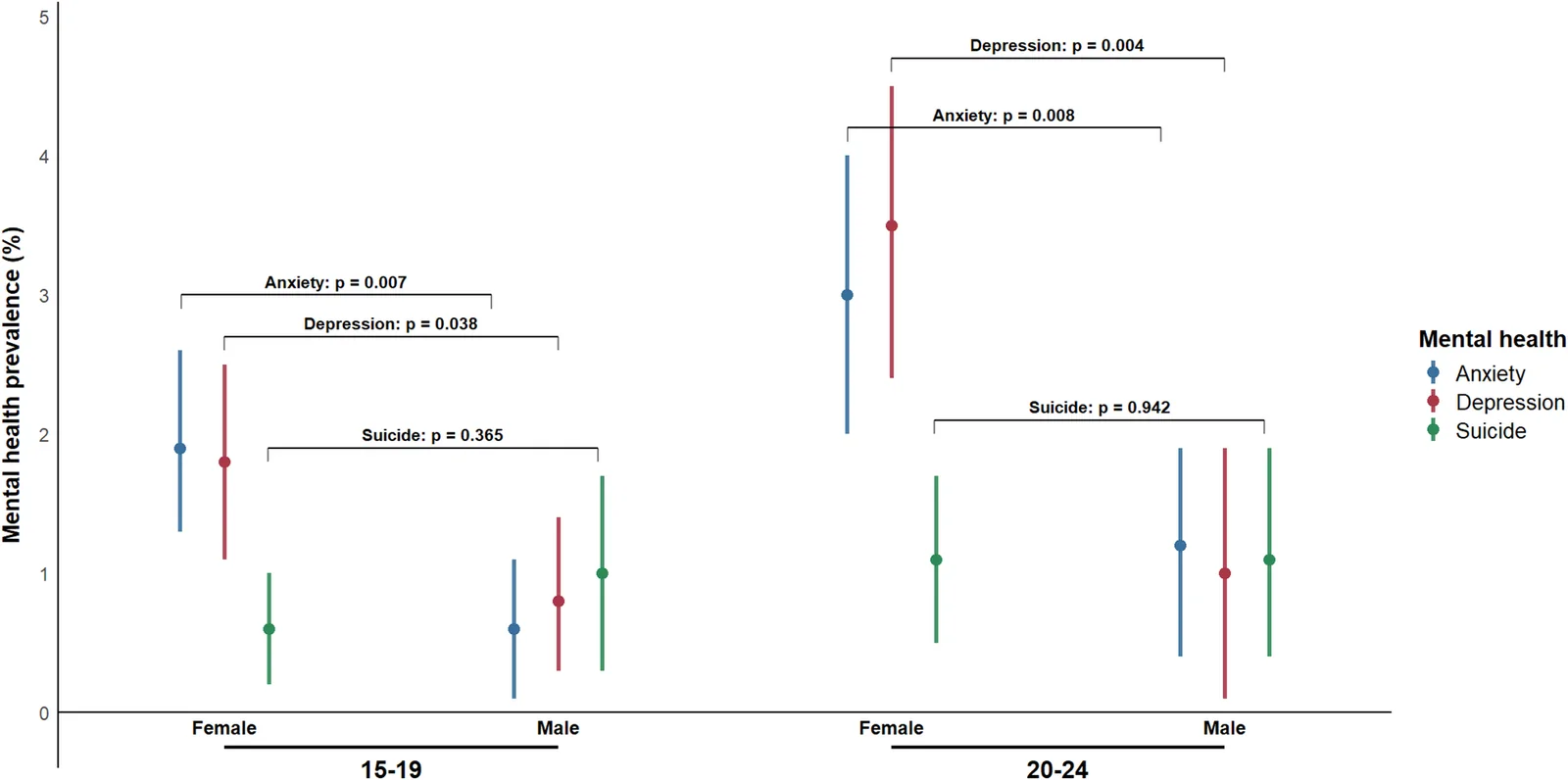
Weighted potential depression, anxiety, and suicidality prevalence in males and females in Kazakhstan, stratified by age. Weighted proportions with 95% confidence intervals (CI). Dots represent the weighted prevalence. Vertical lines indicate 95% CIs. P-values indicate differences between males and females within each age group, calculated using Pearson’s χ² test with Rao & Scott adjustment

**Fig. 2:**
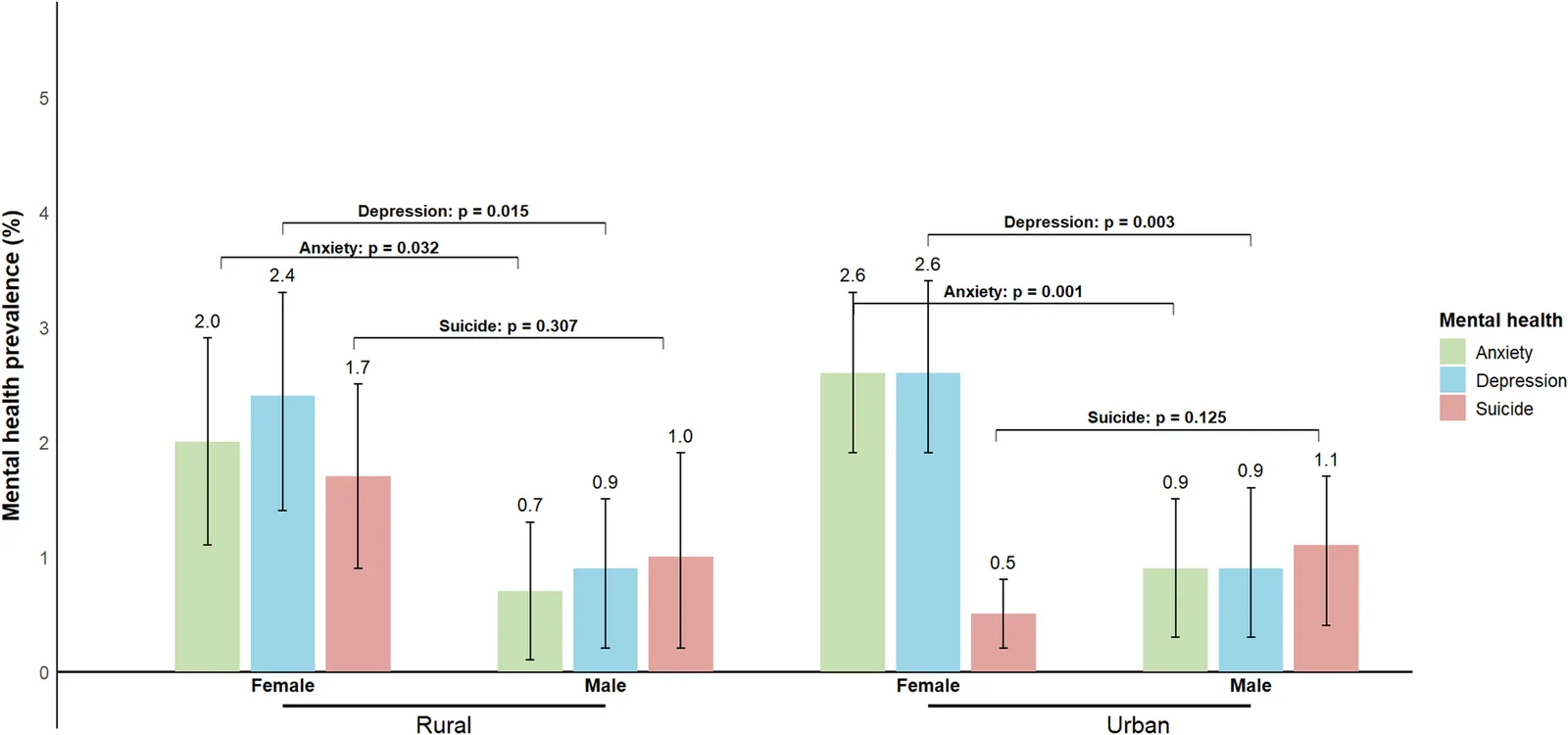
Weighted potential depression, anxiety, and suicidality prevalence in males and females in Kazakhstan, stratified by area of residence. Weighted proportions with 95% confidence intervals (CI). Vertical lines indicate 95% CIs. P-values indicate differences between males and females within each area of residence, calculated using Pearson’s χ² test with Rao & Scott adjustment

Stratified analyses revealed that sex-specific differences in the prevalence of potential depression and anxiety were maintained across age groups (Fig. 1). Among participants aged 15–19 years, females reported a significantly higher prevalence of potential depression (1.8% vs. 0.8% in males; *p* = 0.038) and potential anxiety (1.9% vs. 0.6% in males; *p* = 0.007). This disparity persisted in the 20–24 age group, where potential depression (3.5% in females vs. 1.0% in males; *p* = 0.004) and potential anxiety (3.0% vs. 1.2%) remained significantly more prevalent among female participants. Notably, no significant sex differences in suicidality were identified in either the 15–19 or 20–24 age cohorts. Furthermore, when stratified by area of residence (Fig. 2), females consistently exhibited a higher prevalence of depression and anxiety than males in both rural (depression: *p* = 0.015; anxiety: *p* = 0.032) and urban settings (depression: *p* = 0.003; anxiety: *p* = 0.001).

### Geographic distribution

**Fig. 3:**
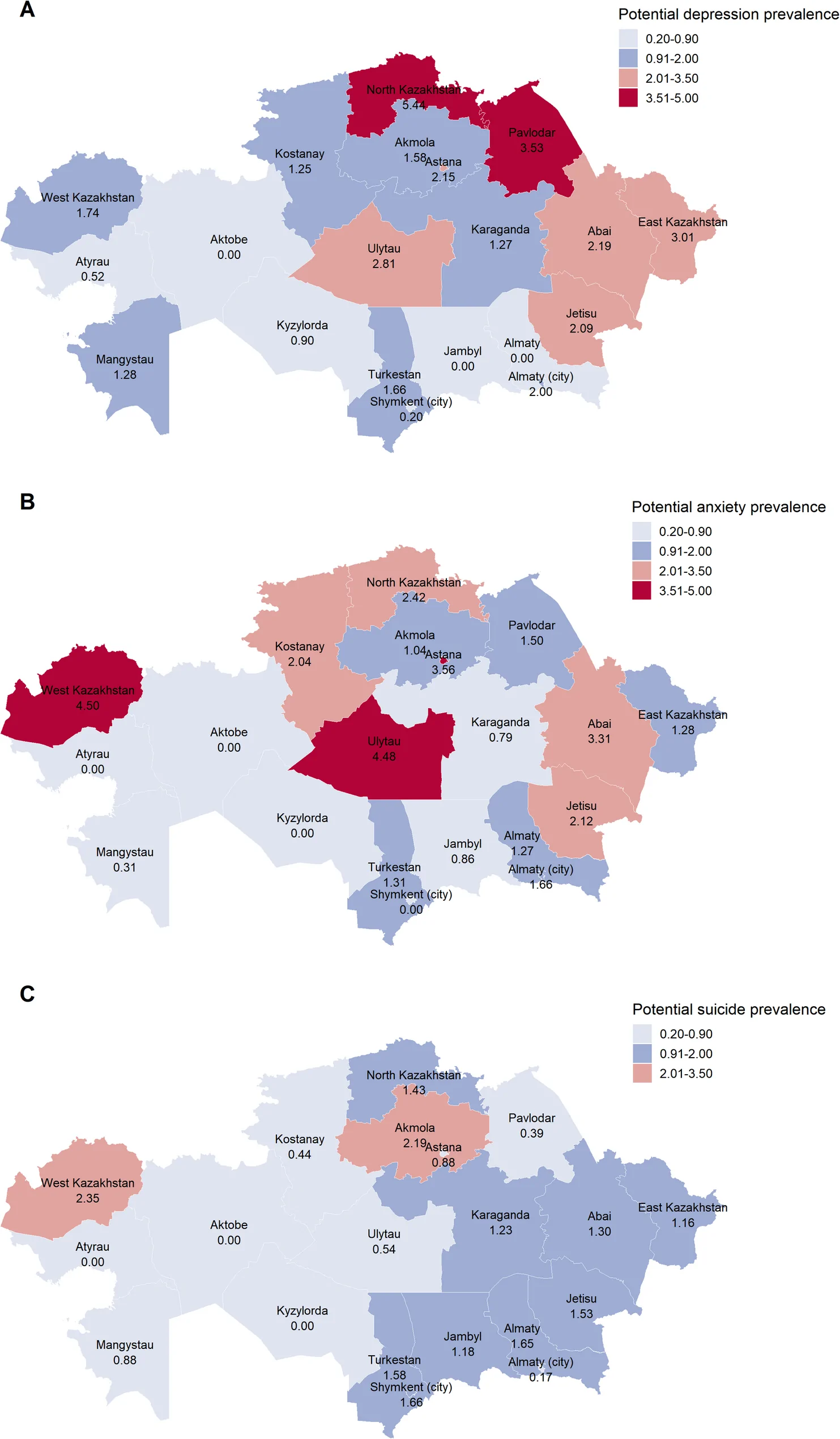
Weighted potential depression, anxiety, and suicidality prevalence by region in Kazakhstan. (**A**) Weighted potential depression prevalence (**B**) Weighted potential anxiety prevalence (**C**) Weighted potential suicidality prevalence. Color shading represents prevalence categories, with darker shades indicating a higher prevalence. Numbers on the map represent the weighted prevalence (%)

Regional prevalence estimates varied significantly across regions for potential depression (*p* = 0.011) and potential anxiety (*p* = 0.002), but not for potential suicidality (*p* = 0.471) (Supplementary Table 3). The highest prevalence of potential depression was documented in North Kazakhstan (5.44%, 95% CI 2.83–10.21), while West Kazakhstan reported the highest prevalence for potential anxiety (4.51%, 95% CI 2.17, 9.12). For suicidality, West Kazakhstan had the highest prevalence (2.35%, 95% CI: 0.84–6.42), although the regional difference was not statistically significant (Fig. 3, Supplementary Table 3).

### Risk factors and population attributable fractions

In unadjusted Poisson regression models (Table 2), female sex (RR = 2.83, 95% CI = 1.61–4.98), being married (RR = 2.11, 95% CI = 1.26–3.52), and having non-Kazakh ethnicity (RR = 1.95, 95% CI = 1.21–3.14) were positively associated with potential depression. After full adjustment for potential confounders (Table 2), only female sex (aRR = 2.72, 95% CI = 1.55–4.79) and non-Kazakh ethnicity (aRR = 1.88, 95% CI = 1.17–3.02) remained significantly associated with potential depression. The PAF for depression was highest for female sex (45.6%), followed by non-Kazakh ethnicity (19.5%).

**Table 2:** The association between sociodemographic factors and potential depression

Variable	Unadjusted RR (95% CI)	*p*-value	Adjusted RR (95% CI)	*p*-value	PAF
Age					
15–19 years	Ref		Ref		
20–24 years	1.77 (1.13, 2.79)	0.013	1.59 (0.90, 2.81)	0.113	19.9%
Sex					
Male	Ref		Ref		
Female	2.83 (1.61, 4.98)	< 0.001	2.72 (1.55, 4.79)	< 0.001	45.6%
Residence					
Rural	Ref		Ref		
Urban	1.14 (0.72, 1.80)	0.573	1.08 (0.62, 1.89)	0.787	5.1%
Education					
College or above	Ref		Ref		
High school and below	0.96 (0.62, 1.49)	0.845	1.16 (0.69, 1.94)	0.577	8.4%
Marital status					
Unmarried	Ref		Ref		
Married	2.11 (1.26, 3.52)	0.004	1.24 (0.68, 2.26)	0.480	3.1%
Wealth					
Middle	Ref		Ref		
Low	1.31 (0.75, 2.29)	0.348	1.26 (0.71, 2.25)	0.425	12.6%
High	1.44 (0.77, 2.72)	0.257	1.44 (0.75, 2.74)	0.270	9.2%
Ethnicity					
Kazakh	Ref		Ref		
Non-Kazakh	1.95 (1.21, 3.14)	0.006	1.88 (1.17, 3.02)	0.009	19.5%

Weighted unadjusted and adjusted risk ratios (RR) with 95% confidence intervals (CI) were estimated using modified Poisson regression models. Adjusted models controlled for age, sex, residence, education, marital status, wealth and ethnicity. PAF was calculated based on the adjusted model; PAF, Population Attributable Fraction; RR, Risk Ratio; Ref, Reference category

For potential anxiety, in the fully adjusted model female sex (aRR = 2.80, 95% CI = 1.58–4.96), non-Kazakh ethnicity (aRR = 1.99, 95% CI = 1.28–3.11), and household wealth status—both lower wealth (aRR = 1.89, 95% CI = 1.00–3.55) and higher wealth (aRR = 2.20, 95% CI = 1.06–4.56)—were identified as significant risk factors (Table 3). Corresponding PAFs indicated that female sex accounted for the largest proportion of attributable risk (46.8%), followed by low wealth (33.0%) and high wealth (21.7%).

**Table 3:** The association between sociodemographic factors and potential anxiety

Variable	Unadjusted RR (95% CI)	*p*-value	Adjusted RR (95% CI)	*p*-value	PAF
Age					
15–19	Ref		Ref		
20–24	1.73 (1.12, 2.68)	0.013	1.48 (0.92, 2.38)	0.107	16.8%
Sex					
Male	Ref		Ref		
Female	2.95 (1.68, 5.17)	< 0.001	2.80 (1.58, 4.96)	< 0.001	46.8%
Residence					
Rural	Ref		Ref		
Urban	1.35 (0.82, 2.22)	0.232	1.26 (0.78, 2.04)	0.336	14.9%
Education					
College or above	Ref		Ref		
High school and below	0.86 (0.55, 1.34)	0.498	1.03 (0.67, 1.59)	0.902	1.7%
Marital status					
Unmarried	Ref		Ref		
Married	1.99 (1.18, 3.36)	0.010	1.22 (0.71, 2.11)	0.474	3.1%
Wealth					
Middle	Ref		Ref		
Low	1.87 (0.98, 3.57)	0.058	1.89 (1.00, 3.55)	0.049	33.0%
High	2.26 (1.10, 4.64)	0.027	2.20 (1.06, 4.56)	0.034	21.7%
Ethnicity					
Kazakh	Ref		Ref		
Non-Kazakh	2.03 (1.28, 3.23)	0.003	1.99 (1.28, 3.11)	0.002	14.9%

Weighted unadjusted and adjusted risk ratios (RR) with 95% confidence intervals (CI) were estimated using modified Poisson regression models. Adjusted models controlled for age, sex, residence, education, marital status, wealth and ethnicity; PAF was calculated based on the adjusted model; PAF, Population Attributable Fraction; RR, Risk Ratio; Ref, Reference category

No significant sociodemographic risk factors were identified for potential suicidality in either unadjusted or adjusted analyses (Table 4).

**Table 4:** The association between sociodemographic factors and potential suicidality

Variable	Unadjusted RR (95% CI)	*p*-value	Adjusted RR (95% CI)	*p*-value	PAF
Age					
15–19	Ref		Ref		
20–24	1.35 (0.73, 2.50)	0.335	1.77 (0.87, 3.58)	0.113	24.4%
Sex					
Female	Ref		Ref		
Male	1.19 (0.63, 2.26)	0.599	1.08 (0.56, 2.11)	0.810	3.9%
Residence					
Urban	Ref		Ref		
Rural	1.67 (0.88, 3.18)	0.120	1.32 (0.68, 2.56)	0.412	9.4%
Education					
College or above	Ref		Ref		
High school and below	1.61 (0.85, 3.07)	0.146	1.67 (0.76, 3.65)	0.200	27.7%
Marital status					
Married	Ref		Ref		
Unmarried	1.09 (0.49, 2.43)	0.830	1.50 (0.66, 3.43)	0.335	30.0%
Wealth					
Middle	Ref		Ref		
Low	2.06 (0.84, 5.06)	0.114	1.80 (0.72, 4.52)	0.207	30.7%
High	0.97 (0.29, 3.22)	0.106	1.02 (0.31, 3.42)	0.972	0.5%
Ethnicity					
Kazakh	Ref		Ref		
Non-Kazakh	1.15 (0.55, 2.41)	0.716	1.09 (0.50, 2.39)	0.831	2.4%

Weighted unadjusted and adjusted risk ratios (RR) with 95% confidence intervals (CI) were estimated using modified Poisson regression models. Adjusted models controlled for age, sex, residence, education, marital status, wealth and ethnicity; PAF was calculated based on the adjusted model; PAF, Population Attributable Fraction; RR, Risk Ratio; Ref, Reference category

Sensitivity analyses (Supplementary Tables 4–7) utilizing unimputed data confirmed the stability and robustness of these primary findings.

## Discussion

### Principal findings

In this nationally representative study that used data from 5,079 young people aged 15–24 years in Kazakhstan, the prevalence of potential depression, anxiety, and suicidality was 1.70%, 1.59%, and 0.96%, respectively. Female sex was the strongest and most consistent correlate of potential depression and anxiety, with nearly threefold higher risks compared with males. Non-Kazakh ethnicity was also associated with higher risks of both conditions, while household wealth showed a U-shaped association with anxiety, with higher anxiety risks observed at both lower and higher levels of household wealth. Subgroup analyses demonstrated a consistently higher prevalence of depression and anxiety among females in both age groups and in urban and rural settings. Geographic analyses further revealed substantial regional variation in these phenomena, with the highest prevalence of depression observed in North Kazakhstan and the highest prevalence of anxiety in West Kazakhstan. In contrast, no sociodemographic factors were significantly associated with suicidality in adjusted analyses.

### Potential explanations and comparison with previous studies

The prevalence of depression and anxiety in our study was lower than worldwide estimates. According to the 2021 Global Burden of Disease Study, the 12-month prevalence of depression and anxiety among 15–24 years was 3.38–4.68% and 5.29–5.66%, respectively [26]. Evidence from diverse settings indicates a substantially higher and more variable burden among children and adolescents, with depression prevalence ranging from approximately 1% to 58% and anxiety from 1% to 30%.[27]. These findings highlight the wide variation in the prevalence of depression and anxiety, especially in resource limited settings, which may reflect differences in screening instruments, diagnostic thresholds, reference period, and cultural context. In Central Asia, persistent stigma surrounding mental health and limited awareness may discourage symptom disclosure [28], potentially leading to underreporting. Consistent with previous studies [29–31], female sex emerged as the most robust and consistent correlate of depression and anxiety. Our results showed that females exhibited nearly a threefold higher risk compared with males, which aligns with earlier research which highlighted that women are about twice as likely as men to develop depression over their lifetime [32]. The higher prevalence of poorer mental health observed among females may be explained by a range of biopsychosocial factors, including gender inequity, a tendency to internalize distress, higher exposure to maltreatment—particularly childhood sexual abuse—and biological factors such as estrogen [14], [33], [34]. As for ethnicity, although direct empirical evidence on ethnic mental health disparities in Kazakhstan remains limited [13], [35], studies from other multiethnic settings suggest that minority populations may experience acculturation stress, discrimination, social marginalization, and reduced access to culturally appropriate mental health services. These factors, along with socio-economic disadvantage and social isolation may further elevate the risk of developing depression and anxiety in these ethnic groups [7], [36]. Furthermore, the PAF estimates provided additional population-level insight into sex- and ethnicity-related differences in mental health outcomes and may help identify groups requiring greater attention in prevention and early identification. Further research is needed to explore the potentially modifiable factors contributing to these disparities. Interestingly, household wealth showed a U-shaped association with anxiety. This pattern may reflect different sources of socioeconomic stress across the wealth spectrum. Previous research has shown that lower socioeconomic status is associated with poorer mental health, with financial hardship and material deprivation potentially acting as persistent stressors that increase vulnerability to anxiety [37]. However, the underlying mechanisms for high-income status may be different from those for lower income status. Evidence suggests that parents with higher socioeconomic status tend to have higher expectations for their children’s achievement [38]. These expectations may increase pressure to achieve, leading to greater worry about performance and fear of disappointing parents, which may contribute to internalizing problems [39], [40].

For suicidality, the prevalence observed in this study (0.9%) was consistent with findings from a national pilot study conducted between 2015 and 2017 in Kazakhstan, which reported suicidal ideation rates of 0.7–1.4% and suicide attempt rates of 0.6–0.7% among adolescents [41]. However, the estimate remains substantially lower than global meta-analytic findings suggesting that 5–17% of young people experienced suicidality [42]. The discrepancy that may be attributable to differences in assessment tools, potential underreporting, variations in the availability and choice of suicide methods, and differences in age stages across study populations [43–45]. Notably, we did not identify significant sex difference or differences in the sociodemographic risk factors for suicidality. This contrasts with previous inconsistent findings on sex differences in suicidal behavior: some studies reported that females were more likely to experience suicidal ideation [29], [45], whereas males have a higher risk of suicide mortality [46], [47]. The absence of significant sociodemographic risk factors for suicidality suggests that suicidal behaviors may not simply be an extension of depression or anxiety. Instead, the factors influencing suicidality may be more complex, involving multidimensional interactions among social, psychological, and life-event-related factors [45], [48]. However, the relatively low prevalence of suicidality may have limited the statistical power to detect significant associations, particularly in the fully adjusted model. Therefore, the lack of statistically significant findings should be interpreted with caution. Future research should incorporate additional psychosocial factors to further explore the risk mechanisms underlying suicidality among young populations.

Subgroup analyses emphasized that the persistent gender disparity in potential depression and anxiety remained evident across both age groups and area of residence, with females consistently showing a higher prevalence than males. This pattern aligns with a growing body of epidemiological evidence demonstrating that females, particularly in late adolescence and early adulthood, have higher rates of depression and anxiety than males [2], [49]. Such sex differences are consistent with the internalizing–externalizing framework of psychopathology, which conceptualizes depression and anxiety as manifestations of a broader internalizing dimension and suggests greater female liability to internalizing symptoms [50]. The emergence and persistence of this sex disparity may reflect the interplay of developmental, biological, and psychosocial processes, including pubertal hormonal changes, differential exposure to stressors, and adverse childhood experiences [32]. Notably, the persistence of these differences across urban and rural settings suggests that gender-specific vulnerabilities operate independently of residential context and may be pervasive across diverse social environments. This robustness may reflect underlying structural and social inequalities, whereby young women experience consistently higher exposure to gender-related stressors and constrained social roles across both urban and rural settings [32]. These results underscore the need for mental health interventions and prevention strategies that are sensitive to gender differences and address the unique vulnerabilities of young females.

Significant regional differences were observed for potential depression and anxiety, with the highest observed prevalence in North Kazakhstan and West Kazakhstan, respectively. No significant regional differences were found for potential suicidality. These variations may reflect underlying geographical heterogeneity in culture, access to health and social services, socioeconomic development and structural inequalities across regions [51], [52]. study showed that North Kazakhstan experienced lower economic development and higher unemployment, potentially exposing residents to chronic stressors that manifest primarily as depression [51]. In contrast, West Kazakhstan is characterized by industrial activity, migration fluctuations, and relative social isolation [52], which may increase exposure to acute stressors, uncertainty, and limited access to mental health services, which could contribute to anxiety. Additionally, cultural factors, including stigma surrounding mental health and variations in help-seeking behavior across regions, and differences in the religious context, may influence the type of symptoms reported and detected in surveys. Religiosity may further shape social norms, stigma, coping strategies, thereby affecting both the experience and reporting of mental health symptoms [53]. Although our study did not directly assess the factors underlying these regional differences, existing literature possible cultural, social, and environmental factors may influence how mental health is perceived and reported. Future research is needed to better understand these contextual factors across diverse settings, which may help inform more targeted regional planning and resource allocation for mental health services.

### Strength and limitations

This study has several strengths. First, it used nationally representative data from the 2024 Kazakhstan Multiple Indicator Cluster Survey (MICS), allowing robust population-level estimates of potential depression, anxiety, and suicidality among young people aged 15–24 years. The standardized multi-stage sampling design and the use of survey weights improve the generalisability of the findings to the national population. Second, validated screening instruments—the Patient Health Questionnaire-2 (PHQ-2) and the Generalized Anxiety Disorder Scale-2 (GAD-2)—were used to assess depressive and anxiety symptoms. These ultra-brief tools have been widely applied in epidemiological studies and have demonstrated acceptable reliability and discriminative validity compared with longer instruments [19], [20]. Third, the relatively large sample size enabled the examination of sociodemographic disparities across multiple population subgroups and allowed estimation of population attributable fractions, providing insights into the relative contribution of demographic factors to mental health outcomes.

Despite this, several limitations should also be considered when interpreting these findings. First, depression and anxiety were measured using brief screening instruments rather than clinical diagnostic interviews, and suicidality was based on self-reported behaviors. Although the PHQ-2 and GAD-2 are validated screening tools, they identify potential symptoms rather than confirmed clinical disorders and may therefore lead to misclassification or underestimation of true prevalence. In addition, reliance on self-reported mental health outcomes may introduce recall bias and underreporting, particularly in cultural contexts where mental health stigma remains strong. Second, although the survey was nationally representative, certain high-risk or populations may be underrepresented, which could further contribute to underestimation of prevalence. In addition, differences between included and excluded participants in marital status and residence may have introduced selection bias, potentially influencing prevalence estimates. Third, the cross-sectional design precludes causal interpretation of the associations observed between sociodemographic characteristics and mental health outcomes. Finally, the analysis was limited to the sociodemographic variables available in the survey dataset. The absence of detailed psychosocial, environmental, and clinical information—such as family environment, trauma exposure, or service access—may have resulted in residual confounding and limits the ability to fully explain the observed patterns.

## Conclusions

In conclusion, this study provides the most comprehensive understanding to date of the epidemiology of potential depression, anxiety, and suicidality among the young population in Kazakhstan. Given that youth mental health has been identified as an important objective within the United Nations Sustainable Development Goals [54], reliable national estimates are essential to allocate resources for treatment and prevention efforts. These findings may inform targeted prevention strategies, particularly for female and ethnic minority youth, while highlighting the importance of strengthening culturally sensitive and accessible mental health services. Future longitudinal and mechanism-oriented studies are warranted to further identify and clarify the range of factors associated with these mental health outcomes and to support the development of more targeted early identification and prevention efforts.

## Supplementary information

Below is the link to the electronic supplementary material.

Supplementary Material 1: Tables S1-S7 and Figure 1.

## Data Availability

This study used data that are available from publicly accessible data sources. Raw datasets are available from UNICEF’s online platform at https://mics.unicef.org/.

## Abbreviations

MICS: Multiple Indicator Cluster Survey
YLDs: Years lived with disability
LMICs: Low- and middle-income countries
UNICEF: United Nations Children’s Fund
CIS: Commonwealth of Independent States
SDG: Sustainable Development Goal
PHQ-2: the 2-item Patient Health Questionnaire
GAD-2: the 2-item Generalized Anxiety Disorder Scale
MICE: Multiple imputation by chained equations
aRRs: Adjusted risk ratios
CIs: Confidence intervals
PAFs: Population attributable fractions
