# Supplementary material for: National prevalence and sociodemographic correlates of potential depression, anxiety, and suicidality among the young population in Kazakhstan: a national population-based study
Source: BMC Psychol. 2026 Oct 1;14:1421. doi: 10.1186/s40359-026-05618-8 (PMC13632261; doi:10.1186/s40359-026-05618-8)
Supplement: Supplementary file 1 [file 40359_2026_5618_MOESM1_ESM.docx]

# **Supplementary appendix**

**Supplementary Figure 1. Study flow chart**

| **Supplementary Table 1. The definition for key variables** | |
| --- | --- |
| Variables | Define |
| The 2-item Patient Health Questionnaire (PHQ-2) | |
| Depressed mood | Participants were asked: “During the past two weeks, how often have you been feeling very sad or depressed, and during the past two weeks, how often have you felt hopeless about the future?” Response options included: (0) never, (1) sometimes, (2) often, (3) always. Depressed mood was defined as the maximum value from the two items. |
| Anhedonia | Participants were asked: “During the past two weeks, how often have you not enjoyed doing things you used to enjoy, such us playing sports, singing and dancing, spending time with friends, watching videos?” Response options included: (0) never, (1) sometimes, (2) often, (3) always. |
| The 2-item Generalized Anxiety Disorder Scale (GAD-2) | |
| Nervous | Participants were asked: “During the past two weeks, how often have you felt nervous, anxious, or on edge?” Response options included: (0) never, (1) sometimes, (2) often, (3) always. |
| Worry | Participants were asked: “During the past two weeks, how often have you felt unable to stop or control your worries?” (0) never, (1) sometimes, (2) often, (3) always. |
| Suicidality | |
| Suicide attempt | In the past 12 months, did you try to harm yourself with the intention or desire to end your life? For example, by cutting yourself on purpose, taking poison or too much medicine, or trying to hang yourself? (1) Yes, (2) No |
| Suicide thought | In the past 2 weeks, have you thought about doing something to end your life? (1) Yes, (2) No |
| Age | 1=15-19 years old 2=20-24 years old |
| Sex | 1=Male 2=Female |
| Education | What is the highest level and grade or year of school has ever attended?  1=High school and below (Primary, preschool or none; Lower secondary; Upper secondary; TVE)  2=College or above (Post secondary; Higher) |
| Marital status | Are you currently married or living together with someone as if married? 1=Married  2=Unmarried (Formerly married; Never married) |
| Residence | Area: 1=Urban area 2=Rural area |
| Wealth | Wealth quintiles represent the relative wealth within a country, and are derived from wealth scores which are calculated based on housing characteristics and both household and personal assets. The original wealth index is typically divided into five quintiles (poorest, second, middle, fourth, and richest).  1=Low (Poorest, Second, Middle) 2=Middle (Fourth) 3=High (Richest) |
| Ethnicity | To which ethnic group does the household head belong 1=Kazakh 2=Non-Kazakh (Russian; Uzbek; Ukrainian; Uyghur; Other) |

| **Supplementary Table 2. Comparison of sociodemographic characteristics between included versus excluded participants aged 15-24 years** | | | |
| --- | --- | --- | --- |
| Variables | Included cases N (%) | Excluded cases N (%) | p-value |
| Overall | 5079 (98.2%) | 54 (1.8%) |  |
| Age |  |  | 0.678 |
| 15-19 | 2891 (58.0%) | 27 (55.4%) |  |
| 20-24 | 2188 (42.0%) | 27 (44.6%) |  |
| Sex |  |  | 0.455 |
| Male | 1790 (51.2%) | 19 (55.9%) |  |
| Female | 3289 (48.8%) | 35 (44.1%) |  |
| Residence |  |  | 0.012 |
| Urban | 3115 (67.6%) | 41 (87.3%) |  |
| Rural | 1964 (32.4%) | 13 (12.7%) |  |
| Education |  |  | 0.386 |
| High school and below | 2848 (57.2%) | 32 (61.6%) |  |
| College or above | 2231 (42.8%) | 22 (38.4%) |  |
| Marital status |  |  | <0.001 |
| Unmarried | 4211 (85.6%) | 36 (70.8%) |  |
| Married | 868 (14.4%) | 18 (29.2%) |  |
| Wealth |  |  | 0.515 |
| Low | 2920 (55.4%) | 30 (67.2%) |  |
| Middle | 1041 (21.5%) | 11 (15.7%) |  |
| High | 1118 (23.1%) | 13 (17.1%) |  |
| Ethnicity |  |  | 0.922 |
| Kazakh | 3738 (72.4%) | 34 (73.4%) |  |
| Non-Kazakh | 1341 (27.6%) | 20 (26.6%) |  |
| Data are n (unweighted); Weighted percentages (%) were calculated using survey weights; p-value were calculated based on Pearson’s χ² test with Rao & Scott adjustment for categorical variables. | | | |

| **Supplementary Table 3. Regional differences in the prevalence of potential depression, potential anxiety, and suicidality in Kazakhstan** | | | | | | | | | | | |
| --- | --- | --- | --- | --- | --- | --- | --- | --- | --- | --- | --- |
|  | Depression | |  |  | Anxiety | |  |  | Suicidality | |  |
|  | No depression | Depression | p-value |  | No anxiety | Anxiety | p-value |  | No suicidality | Suicidality | p-value |
| Region |  |  | 0.011 |  |  |  | 0.002 |  |  |  | 0.471 |
| Abai | 97.81% (95.35, 98.99) | 2.19% (1.01, 4.65) |  |  | 96.69% (93.61, 98.31) | 3.31% (1.69, 6.39) |  |  | 98.70% (94.44, 99.71) | 1.30% (0.29, 5.56) |  |
| Akmola | 98.42% (94.65, 99.54) | 1.58% (0.46, 5.35) |  |  | 98.96% (96.99, 99.64) | 1.04% (0.36, 3.01) |  |  | 97.81% (93.80, 99.25) | 2.19% (0.75, 6.20) |  |
| Aktobe | 100% (n/a) | 0% (n/a) |  |  | 100% (n/a) | 0% (n/a) |  |  | 100% (n/a) | 0% (n/a) |  |
| Almaty | 100% (n/a) | 0% (n/a) |  |  | 98.73% (95.39, 99.66) | 1.27% (0.34, 4.61) |  |  | 98.35% (95.38, 99.42) | 1.65% (0.58, 4.62) |  |
| Atyrau | 99.48% (97.73, 99.88) | 0.52% (0.12, 2.27) |  |  | 100% (n/a) | 0% (n/a) |  |  | 100% (n/a) | 0% (n/a) |  |
| West Kazakhstan | 98.26% (94.53, 99.46) | 1.74% (0.54, 5.47) |  |  | 95.49% (90.88, 97.83) | 4.51% (2.17, 9.12) |  |  | 97.65% (93.58, 99.16) | 2.35% (0.84, 6.42) |  |
| Jambyl | 100% (n/a) | 0% (n/a) |  |  | 99.13% (97.55, 99.70) | 0.87% (0.30, 2.45) |  |  | 98.82% (95.41, 99.71) | 1.18% (0.29, 4.59) |  |
| Jetisu | 97.91% (92.84, 99.41) | 2.09% (0.59, 7.16) |  |  | 97.88% (94.89, 99.14) | 2.12% (0.86, 5.11) |  |  | 98.47% (93.45, 99.66) | 1.53% (0.34, 6.55) |  |
| Karaganda | 98.73% (95.85, 99.62) | 1.27% (0.38, 4.15) |  |  | 99.21% (96.37, 99.83) | 0.79% (0.17, 3.63) |  |  | 98.78% (94.60, 99.73) | 1.22% (0.27, 5.40) |  |
| Kosatanty | 98.75% (96.44, 99.57) | 1.25% (0.43, 3.56) |  |  | 97.97% (94.71, 99.23) | 2.03% (0.77, 5.29) |  |  | 99.56% (97.76, 99.92) | 0.44% (0.08, 2.24) |  |
| Kyzylorda | 99.10% (93.66, 99.88) | 0.90% (0.12, 6.34) |  |  | 100% (n/a) | 0% (n/a) |  |  | 100% (n/a) | 0% (n/a) |  |
| Mangystau | 98.72% (96.82, 99.49) | 1.28% (0.51, 3.18) |  |  | 99.69% (98.59, 99.93) | 0.31% (0.07, 1.41) |  |  | 99.12% (97.19, 99.73) | 0.88% (0.27, 2.81) |  |
| Pavlodar | 96.47% (92.42, 98.40) | 3.53% (1.60, 7.58) |  |  | 98.50% (95.96, 99.45) | 1.50% (0.55, 4.04) |  |  | 99.61% (97.14, 99.95) | 0.39% (0.05, 2.86) |  |
| North Kazakhstan | 94.56% (89.79, 97.17) | 5.44% (2.83,10.21) |  |  | 97.58% (94.85, 98.88) | 2.42% (1.12, 5.15) |  |  | 98.57% (94.38, 99.65) | 1.43% (0.35, 5.62) |  |
| Turkistan | 98.34% (95.92, 99.34) | 1.66% (0.66, 4.08) |  |  | 98.69% (95.56, 99.62) | 1.31% (0.38, 4.44) |  |  | 98.42% (95.40, 99.47) | 1.58% (0.53, 4.60) |  |
| Ulytau | 97.19% (94.29, 98.64) | 2.81% (1.36, 5.71) |  |  | 95.52% (90.35, 97.98) | 4.48% (2.02, 9.65) |  |  | 99.46% (97.91, 99.86) | 0.54% (0.14, 2.09) |  |
| East Kazakhstan | 96.99% (93.21, 98.69) | 3.01% (1.31, 6.79) |  |  | 98.72% (95.68, 99.63) | 1.28% (0.37, 4.32) |  |  | 98.84% (96.57, 99.61) | 1.16% (0.39, 3.43) |  |
| Astana | 97.85% (95.01, 99.09) | 2.15% (0.91, 4.99) |  |  | 96.44% (92.90, 98.25) | 3.56% (1.75, 7.10) |  |  | 99.12% (96.17, 99.80) | 0.88% (0.20, 3.83) |  |
| Almaty city | 98.00% (96.31, 98.93) | 2.00% (1.07, 3.69) |  |  | 98.34% (96.65, 99.18) | 1.66% (0.82, 3.35) |  |  | 99.83% (98.74, 99.98) | 0.17% (0.02, 1.26) |  |
| Shymkent city | 99.80% (98.44, 99.97) | 0.20% (0.03, 1.56) |  |  | 100% (n/a) | 0% (n/a) |  |  | 98.34% (94.51, 99.51) | 1.66% (0.49, 5.49) |  |
| Weighted percentages (%) were calculated using survey weights, with 95% confidence intervals (CI); % are calculated with row totals as the denominator. p-value were calculated based on Pearson’s χ² test with Rao & Scott adjustment for categorical variables. n/a, 95% CI not estimable due to zero observed cases. | | | | | | | | | | | |

| **Supplementary Table 4. Sample weighted mental health prevalence for various sociodemographic characteristics, using no imputed data** | | | |
| --- | --- | --- | --- |
| Variables | Depression, % (95%CI) | Anxiety, % (95%CI) | Suicide, % (95%CI) |
| Total | 1.70% (1.32-2.09) | 1.60% (1.23-1.96) | 0.96% (0.65-1.28) |
| Age |  |  |  |
| 15-19 | 1.28% (0.86-1.71) | 1.22% (0.82-1.62) | 0.84% (0.44-1.24) |
| 20-24 | 2.28% (1.57-2.99) | 2.11% (1.47-2.75) | 1.13% (0.65-1.61) |
| Sex |  |  |  |
| Male | 0.90% (0.42-1.37) | 0.82% (0.39-1.25) | 1.04% (0.52-1.57) |
| Female | 2.54% (1.96-3.13) | 2.41% (1.85-2.97) | 0.88% (0.53-1.22) |
| Residence |  |  |  |
| Urban | 1.77% (1.27-2.27) | 1.74% (1.27-2.22) | 0.79% (0.42-1.16) |
| Rural | 1.55% (0.99-2.11) | 1.29% (0.76-1.82) | 1.32% (0.74-1.91) |
| Education |  |  |  |
| High school and below | 1.67% (1.13-2.21) | 1.49% (1.04-1.94) | 1.15% (0.70-1.60) |
| College or above | 1.74% (1.22-2.27) | 1.74% (1.15-2.33) | 0.71% (0.33-1.10) |
| Marital status |  |  |  |
| Unmarried | 1.47% (1.08-1.85) | 1.74% (1.15-2.33) | 0.97% (0.62-1.32) |
| Married | 3.09% (1.73-4.46) | 2.78% (1.52-4.05) | 0.89% (0.26-1.53) |
| Wealth |  |  |  |
| Low | 1.75% (1.20-2.29) | 1.68% (1.21-2.16) | 1.25% (0.79-1.72) |
| Middle | 1.34% (0.72-1.96) | 0.90% (0.38-1.43) | 0.61% (0.08-1.13) |
| High | 1.93% (1.08-2.78) | 2.03% (1.07-2.99) | 0.59% (0.10-1.09) |
| Ethnicity |  |  |  |
| Kazakh | 1.35% (0.96-1.74) | 1.24% (0.87-1.62) | 0.93% (0.57-1.28) |
| Non-Kazakh | 2.62% (1.65-3.60) | 2.52% (1.64-3.41) | 1.06% (0.38-1.74) |
| Weighted percentages (%) were calculated using survey weights, with 95% confidence intervals (CI). | | | |

| **Supplementary Table 5. The association between sociodemographic factors and depression, using no imputed data** | | | |
| --- | --- | --- | --- |
| Variables | Adjusted RR (95% CI) | p-value | PAF |
| Age |  |  |  |
| 15-19 | Ref |  |  |
| 20-24 | 1.59 (0.90, 2.81) | 0.113 | 19.9% |
| Sex |  |  |  |
| Male | Ref |  |  |
| Female | 2.73 (1.55, 4.79) | <0.001 | 45.7% |
| Residence |  |  |  |
| Rural | Ref |  |  |
| Urban | 1.08 (0.62, 1.89) | 0.787 | 5.1% |
| Education |  |  |  |
| College or above | Ref |  |  |
| High school and below | 1.16 (0.69, 1.94) | 0.578 | 8.4% |
| Marital status |  |  |  |
| Unmarried | Ref |  |  |
| Married | 1.24 (0.68, 2.26) | 0.481 | 3.3% |
| Wealth |  |  |  |
| Middle | Ref |  |  |
| Low | 1.27 (0.71, 2.25) | 0.422 | 13.0% |
| High | 1.44 (0.75, 2.75) | 0.269 | 9.2% |
| Ethnicity |  |  |  |
| Kazakh | Ref |  |  |
| Non-Kazakh | 1.88 (1.17, 3.01) | 0.009 | 19.5% |
| Adjusted risk ratios (RR) with 95% confidence intervals (CI) were estimated using modified Poisson regression models; Adjusted models controlled for age, sex, residence, education, marital status, wealth, and ethnicity; PAF was calculated based on the fully adjusted model; Population Attributable Fraction, PAF; RR, Risk Ratio. | | | |

| **Supplementary Table 6. The association between sociodemographic factors and anxiety, using no imputed data** | | | |
| --- | --- | --- | --- |
|  | Adjusted RR (95% CI) | p-value | PAF |
| Age |  |  |  |
| 15-19 | Ref |  |  |
| 20-24 | 1.48 (0.92, 2.39) | 0.105 | 16.8% |
| Sex |  |  |  |
| Male | Ref |  |  |
| Female | 2.80 (1.58, 4.97) | <0.001 | 46.8% |
| Residence |  |  |  |
| Rural | Ref |  |  |
| Urban | 1.27 (0.78, 2.04) | 0.334 | 15.4% |
| Education |  |  |  |
| College or above | Ref |  |  |
| High school and below | 1.03 (0.67, 1.59) | 0.895 | 1.7% |
| Marital status |  |  |  |
| Unmarried | Ref |  |  |
| Married | 1.22 (0.70, 2.11) | 0.479 | 3.1% |
| Wealth |  |  |  |
| Middle | Ref |  |  |
| Low | 1.89 (1.00, 3.55) | 0.049 | 33.0% |
| High | 2.19 (1.06, 4.54) | 0.035 | 21.6% |
| Ethnicity |  |  |  |
| Kazakh | Ref |  |  |
| Non-Kazakh | 1.99 (1.28, 3.10) | 0.002 | 21.5% |
| Adjusted risk ratios (RR) with 95% confidence intervals (CI) were estimated using modified Poisson regression models; Adjusted models controlled for age, sex, residence, education, marital status, wealth, and ethnicity; PAF was calculated based on the fully adjusted model; Population Attributable Fraction, PAF; RR, Risk Ratio. | | | |

| **Supplementary Table 7. The association between sociodemographic factors and suicidality, using no imputed data** | | | |
| --- | --- | --- | --- |
| Variables | Adjusted RR (95% CI) | p-value | PAF |
| Age |  |  |  |
| 15-19 | Ref |  |  |
| 20-24 | 1.76 (0.87, 3.57) | 0.114 | 24.2% |
| Sex |  |  |  |
| Female | Ref |  |  |
| Male | 1.09 (0.56, 2.11) | 0.810 | 4.4% |
| Residence |  |  |  |
| Urban | Ref |  |  |
| Rural | 1.32 (0.68, 2.56) | 0.408 | 9.4% |
| Education |  |  |  |
| College or above | Ref |  |  |
| High school and below | 1.67 (0.76, 3.65) | 0.200 | 27.7% |
| Marital status |  |  |  |
| Married | Ref |  |  |
| Unmarried | 1.50 (0.66, 3.43) | 0.335 | 30.0% |
| Wealth |  |  |  |
| Middle | Ref |  |  |
| Low | 1.81 (0.72, 4.52) | 0.207 | 31.0% |
| High | 1.02 (0.31, 3.43) | 0.968 | 0.5% |
| Ethnicity |  |  |  |
| Kazakh | Ref |  |  |
| Non-Kazakh | 1.09 (0.50, 2.39) | 0.831 | 2.4% |
| Adjusted risk ratios (RR) with 95% confidence intervals (CI) were estimated using modified Poisson regression models; Adjusted models controlled for age, sex, residence, education, marital status, wealth, and ethnicity; PAF was calculated based on the fully adjusted model; Population Attributable Fraction, PAF; RR, Risk Ratio. | | | |
